# Cyclooxygenase-2 Expression in Bladder Cancer and Patient Prognosis: Results from a Large Clinical Cohort and Meta-Analysis

**DOI:** 10.1371/journal.pone.0045025

**Published:** 2012-09-13

**Authors:** Maciej J. Czachorowski, André F. S. Amaral, Santiago Montes-Moreno, Josep Lloreta, Alfredo Carrato, Adonina Tardón, Manuel M. Morente, Manolis Kogevinas, Francisco X. Real, Núria Malats, for the SBC/EPICURO investigators

**Affiliations:** 1 Genetic and Molecular Epidemiology Group, Spanish National Cancer Research Centre (CNIO), Madrid, Spain; 2 Servicio de Anatomía Patológica, Hospital Universitario Marqués de Valdecilla, Santander, Spain; 3 Institut Municipal d’Investigació Mèdica – Hospital del Mar, Barcelona, Spain; 4 Departament de Patologia, Hospital del Mar – Parc de Salut Mar, Barcelona, Spain; 5 Departament de Ciències Experimentals i de la Salut, Universitat Pompeu Fabra, Barcelona, Spain; 6 Hospital General Universitario de Elche, Elche, Spain; 7 Hospital Ramon y Cajal, Madrid, Spain; 8 Universidad de Oviedo, Oviedo, Spain; 9 Tumor Bank Unit, Spanish National Cancer Research Centre (CNIO), Madrid, Spain; 10 Centre de Recerca en Epidemiologia Ambiental (CREAL), Barcelona, Spain; 11 Epithelial Carcinogenesis Group, Spanish National Cancer Research Centre (CNIO), Madrid, Spain; University of California Irvine, United States of America

## Abstract

Aberrant overexpression of cyclooxygenase-2 (COX2) is observed in urothelial carcinoma of the bladder (UCB). Studies evaluating COX2 as a prognostic marker in UCB report contradictory results. We determined the prognostic potential of COX2 expression in UCB and quantitatively summarize the results with those of the literature through a meta-analysis. Newly diagnosed UCB patients recruited between 1998–2001 in 18 Spanish hospitals were prospectively included in the study and followed-up (median, 70.7 months). Diagnostic slides were reviewed and uniformly classified by expert pathologists. Clinical data was retrieved from hospital charts. Tissue microarrays containing non-muscle invasive (n = 557) and muscle invasive (n = 216) tumours were analyzed by immunohistochemistry using quantitative image analysis. Expression was evaluated in Cox regression models to assess the risk of recurrence, progression and disease-specific mortality. Meta-hazard ratios were estimated using our results and those from 11 additional evaluable studies. COX2 expression was observed in 38% (211/557) of non-muscle invasive and 63% (137/216) of muscle invasive tumors. Expression was associated with advanced pathological stage and grade (p<0.0001). In the univariable analyses, COX2 expression - as a categorical variable - was not associated with any of the outcomes analyzed. As a continuous variable, a weak association with recurrence in non-muscle invasive tumors was observed (p-value = 0.048). In the multivariable analyses, COX2 expression did not independently predict any of the considered outcomes. The meta-analysis confirmed these results. We did not find evidence that COX2 expression is an independent prognostic marker of recurrence, progression or survival in patients with UCB.

## Introduction

Urothelial carcinoma of the bladder (UCB) is the most common bladder cancer type in developed nations [1]. UCB predominantly manifests (70–80% of patients) as a non-muscle invasive tumor (NMIBC: pTa-pT1) characterized by an overall good prognosis following transurethral resection in patients with low-grade tumors (pTaG1/2), and intravesical chemotherapy and/or Bacillus Calmette Guerin (BCG) instillation in patients with high-grade tumors (pTaG3 or pT1G2/3) [2]. Approximately 70% of NMIBC patients suffer a recurrence following treatment and a further 15% progress, developing new tumors exhibiting muscle invasion (MIBC: pT2-pT4); the risk of progression being higher among patients with high-grade tumors [2]. Due to a high rate of recurrence and the need for close follow-up over a patient’s lifetime, UCB remains one of the most expensive tumors to treat on a per patient basis [3]. A lower proportion (20–30%) of UCB patients are diagnosed with muscle invasive tumors (MIBC; pT2-pT4) characterized by poor prognosis: 50% of these patients die from their cancer [2]. Genomic profiling and gene expression analyses indicate a strong correlation between these pathologic classifications and the underlying molecular architecture of UCB [4].

Growing evidence indicates that chronic inflammation may increase the risk of UCB [5]. Studies investigating the prolonged use of cyclooxygenase-2 (COX2) inhibiting non-steroidal anti-inflammatory drugs (NSAIDs) have reported a decrease in UCB risk [6], [7]. COX2 is a prostaglandin endoperoxide synthetase that catalyzes the production of prostanoids upon induction by proinflammatory cytokines, growth factors, tumor promoters and other external stimuli [8]. COX2 activation mediates cellular processes also implicated in carcinogenesis such as angiogenesis, cell survival/proliferation and apoptosis [9]. Moreover, studies have shown that bladder tissue from patients with cystitis or UCB exhibits elevated COX2 levels in contrast to benign bladder tissue [10], [11].

While numerous groups have investigated the prognostic potential of COX2 expression in UCB [12]–[31], there is no clear consensus on its utility. The objective of this study was to assess whether COX2 protein expression in UCB cells is associated with prognosis using a large and standardized cohort of newly diagnosed bladder cancer patients. A meta-analysis was also done to summarize these results together with those from other studies published on the topic.

## Materials and Methods

### Study Population

A total of 773 newly diagnosed UCB cases aged 22–80 years (mean ± SD = 66±10 yrs) with a median follow-up of 70.7 months (range 0.7–117.7 months) and available tumor tissue were used in the current analysis. All cases were recruited between 1998 and 2001 from 18 hospitals in five regions of Spain as part of the Spanish Bladder Cancer (SBC)/EPIdemiology of Cancer of the UROthelium (EPICURO) study, a hospital-based case-control study described previously [32]. A pathologist review panel uniformly classified the T stage and grade (G) of each tumor biopsy according to the criteria of the TNM classification and the WHO-ISUP [33], using the three grade redefinition provided by the WHO [34], [35]. All bladder tumor samples used in the study were collected prior to the administration of any intravesical or systemic therapy. Clinical information related to diagnostic procedures, tumor characteristics and treatment was collected from medical records, and a computerized questionnaire was used for the collection of sociodemographic data. NMIBCs were removed by transurethral resection and patients received intravesical chemo- or immunotherapy (i.e. BCG) as appropriate. The majority of patients presenting with MIBCs were treated by radical cystectomy; in cases where surgery was not possible, radiotherapy or systemic chemotherapy were administered. Follow-up information was collected annually from hospital records and through direct telephone interviews by trained monitors using structured questionnaires. Among NMIBCs, recurrence was defined as the appearance of a new NMIBC following a previous negative follow-up cystoscopy, and progression, as the development of a MIBC. In patients initially presenting with MIBCs, any tumor reappearance after treatment was considered progression, regardless of whether the tumor relapse was local or distal. Tumour-specific survival was assessed only for patients with MIBCs. Informed written consent was obtained from study participants in accordance with the Ethics Committees of each participating hospital.

### Immunohistochemistry

Tissue blocks of formalin-fixed, paraffin-embedded primary bladder tumors were used to construct tissue microarrays (TMA) containing tumor cores of 0.6-mm in diameter represented in duplicate and selected from the most representative regions of the tumor on which T and G were based. After deparaffinisation and heat-induced antigen retrieval, all slides were stained simultaneously at the Histology and Immunohistochemistry Core Unit of the CNIO using the PT LINK system as per manufacturer’s instructions (Dako Inc., Glostrup, Denmark). Briefly, tissue sections were incubated with anti-COX2 rabbit monoclonal antibody (ThermoFisher Scientific, Fremont, CA, USA; #RM-9121-R7; pre-diluted, ready-to-use) at room temperature, followed by visualization using the EnVision Flex Visualization system (Dako Inc., Glostrup, Denmark) and exposure to diaminobenzedine. Tissues were then counterstained with haematoxylin, dehydrated and mounted. A section of colon tissue was used as a positive control.

### Evaluation of COX2 Immunostaining

COX2 expression was quantified using the Ariol SL-50 (version 3.1.2, Applied Imaging Corp., San Jose, CA, USA) high-throughput slide imaging scanner. All cores were imaged and processed using a light microscope and the accompanying TMA Multistain Imaging software. The program was trained by a pathologist (SM) to maximize the inclusion of positively stained tumor epithelium while minimizing stromal material, as described previously [36]. COX2 expression score was calculated as the product of the mean intensity of staining (by defining the background and saturation limits of the antibody and imaging sensor, respectively) and the proportion of cellular antibody-positive area divided by total cellular area. Values from replicate cores were averaged to provide a final expression score for each patient. Furthermore, one randomly selected TMA (representing 10% of all cores) was analyzed by direct visual microscopic inspection by an independent pathologist (MMM) to enable comparison with the automated scoring approach. The pathologist-derived score was calculated as the product of COX2 staining intensity (1 = weak, 2 = intermediate, 3 = strong) and a quartile of the percentage of epithelial tumor cells stained (0–4; with 0 representing 0% staining), providing a final categorical score in the range of 0–12. There was a high and significant correlation between the machine and pathologist derived scores (Spearman rho = 0.85; 95%CI = 0.79–0.90; p-value<0.00001). COX2 expression was analyzed as both a continuous variable and categorical variables partitioned at the median and extreme tertiles. Additionally, expression was examined as a categorical variable dichotomized at a threshold (0.340 arbitrary units [au]) above which COX2 expression was considered to be *positive*. This expression threshold was derived by comparing the pathologist’s (MMM) binary assignment of positive expression (i.e. score of 0 vs. score ≥1, as described above) to the machine-derived continuous score using receiver operating characteristic (ROC) curve analysis (area under the curve = 0.95; 86% sensitivity and 92% specificity) [37].

### Meta-analysis

The meta-analysis included COX2 expression results from our own series (using the ROC-derived categorical expression variable) and relevant studies published before 1 January 2012 identified by searching PubMed and ISI Web of Knowledge. The search string used was: (cox2 OR cox-2 OR cyclooxygenase-2 OR "cyclooxygenase 2" OR ptgs2) AND (prognos* OR survival OR mortality OR recurrence OR relapse OR progression) AND ("bladder cancer"). Studies were considered eligible if: (i) they reported the effect measure (as HRs, survival curves or log-rank p-values) of COX2 protein expression on recurrence, progression or disease-specific survival; (ii) COX2 was assessed in primary tumors exhibiting homogeneity in tumor histology (≥75% UCB), and subphenotype (≥75% NMIBC *or* MIBC); (iii) they were written in English or Spanish (Table S1). Reviews, abstracts, non-clinical studies, and duplicate publications were excluded. HRs and 95%CIs were directly extracted from the publications whenever available. For those reporting only the log-rank p-value or the Kaplan–Meier survival curves, the HRs and 95%CIs were independently calculated by two of the co-authors (MJC, AFSA) using the spreadsheet prepared by Sydes and Tierney with any discrepancies resolved by discussion [38]. In a few indicated cases, authors were directly contacted for clarification or provision of data not shown in the published manuscripts (Table S1). The level of heterogeneity among studies was calculated by means of the I^2^ statistic [39], and publication bias was assessed by analyzing funnel plots and Egger’s asymmetry test [40].

### Statistical Analysis

Associations between demographic and clinico-pathological parameters and COX2 expression were assessed using Fisher’s exact test. In NMIBCs, expression was also assessed distinctly in low-grade/risk (pTaG1/G2) and high-grade/risk (pTa/pT2G3) tumors, based on our previous evidence suggesting differential prognostic, genetic and molecular profiles between these subgroups [41], [42]. Recurrence-free, progression-free, and overall disease-specific survival curves were generated using the Kaplan-Meier method, with statistical significance assessed using the log-rank test. Time to each endpoint was calculated from date of primary treatment to the date of event, date of last follow-up, or date of patient’s death. Individuals who did not present any event until the end of the study, those lost to follow-up, or those who died from other causes were censored either at the time of last medical visit or at death. Time to recurrence and progression were defined by applying the “mid-time” between the date of the previous disease-free visit and that when a new event was diagnosed. Survival time was measured as the time from initial treatment to death resulting from cancer. Univariable and multivariable Cox-proportional hazards analysis was used to calculate hazard ratios (HR) and 95% confidence intervals (CI). Schoenfeld residual analysis did not suggest any departure from the proportional hazards assumption in multivariable models.

All statistical analyses were done using STATA (version 10.1 SE, StataCorp, College Station, TX, USA). Statistical tests were two-sided and p-values less than 0.05 were considered significant. The REMARK [43] guidelines for prognostic studies as well as the PRISMA [44] guidelines for systematic reviews and meta-analyses were adhered to in the preparation of the manuscript.

## Results

### Patients and COX2 Expression in Bladder Cancer TMAs

COX2 expression was assessed in 557 patients with NMIBCs and 216 individuals with MIBCs. Median COX2 expression was 0.121 au (range 0–42.590; interquartile range 1.382) in NMIBCs, and 0.760 au (0–30.806; 3.600) in MIBCs (p-value = 4×10^−12^). Representative COX2 immunostaining patterns in UCBs are shown in Figure S1. Of patients with NMIBCs, 41% (230/557) were treated only by transurethral resection, with the remainder (56%) receiving endovesical BCG immunotherapy and/or chemotherapy following transurethral resection, or other treatment (3%; Table 1). Nearly half (46%) of patients with MIBCs were treated by cystectomy, with the remainder receiving systemic chemotherapy, radiotherapy, superficial or other treatment, or some combination thereof (Table 2).

**Table 1:** Distribution of characteristics of patients with NMIBCs by COX2 expression.

			COX2 expression*		
Patient characteristics	Total, N		negative,n	positive,n	P value†
	557		346	211	
**Area**					0,506
** Barcelona**	98		68	30	
** Valles**	105		66	39	
** Elche**	51		32	19	
** Tenerife**	122		71	51	
** Asturias**	181		109	72	
**Age (yrs.)**					0,385
** ≤60**	140		81	59	
** >60 and ≤70**	210		130	80	
** >70**	207		135	72	
**Gender**					0,891
** Men**	494		306	188	
** Women**	63		40	23	
**Tumor Invasion**					<0,0001
** Ta**	477		277	200	
** T1**	80		69	11	
**Grade**					<0,0001
** GI**	200		131	69	
** GII**	219		95	124	
** GIII**	138		120	18	
**Low/High Grade**					<0,0001
** Low (TaG1/TaG2)**	408		221	187	
** High (TaG3/T1G2/T1G3)**	149		125	24	
**Number of tumors**					0,106
** 1**	348		209	139	
** >1**	178		120	58	
** missing**	31		17	14	
**Tumour Size**					0,564
** ≤3 cm**	294		188	106	
** >3 cm**	111		67	44	
** missing**	152		91	61	
**Number of Recurrences**					0,409
** none**	366		232	134	
** at least 1**	191		114	77	
**Treatment** ‡					0,393
** TUR**	230		133	97	
** TUR+BCG**	158		105	53	
** TUR+Chem.**	132		83	49	
** TUR+BCG+Chem.**	19		14	5	
** Other**	18		11	7	

* COX2 expression score dichotomised at the threshold of positivitiy (0,340 au).

† Fisher’s exact test comparing distribution of COX-2 negative versus positive patients; missing values excluded from analysis where applicable.

‡ TUR: transurethral resection; BCG: Bacillus Calmette-Guerin instillation; Chem.: chemotherapy via endovesical instillation.

**Table 2:** Distribution of characteristics of patients with MIBCs by COX2 expression.

			COX2 expression*							
Patient Characteristics	Total, N		negative, N			positive, N		P value†		
	216		79			137				
**Area**									0,207	
** Barcelona**	39		16			23				
** Valles**	36		9			27				
** Elche**	15		9			6				
** Tenerife**	39		14			25				
** Asturias**	87		31			56				
**Gender**									0,816	
** Men**	194		72			122				
** Women**	22		7			15				
**Age (yrs.)**									0,426	
** ≤60**	45		14			31				
** >60 and ≤70**	84		35			49				
** >70**	87		30			57				
**Tumor invasion**									0,896	
** T2**	114		42			72				
** T3**	55		21			34				
** T4**	47		16			31				
**Grade**									0,326	
** GII**	19		9			10				
** GIII**	197		70			127				
**Metastases**									0,296	
** M0**	168		57			111				
** M1**	29		13			16				
** Mx**	19		9			10				
**Lymphatic invasion**									0,862	
** N0**	141		50			91				
** N1, N3**	49		16			33				
** Nx**	26		13			13				
**Number of tumors**									0,008	
** 1**	146		63			83				
** >1**	54		12			42				
** missing**	16		4			12				
**Tumour size**									0,572	
** ≤3 cm**	53		19			34				
** >3 cm**	66		28			38				
** missing**	97		32			65				
**Treatment** ‡									0,417	
** Cystectomy**	67		19			48				
** Cystectomy+Chem.**	32		15			17				
** Chem. only**	23		9			14				
** RT +/− Chem.**	19		7			12				
** Superficial Treatment**	13		3			10				
** Others**	61		25			36				
** missing**	1		1			0				

* COX2 expression score dichotomised at the threshold of positivity (0,340 au).

† Fisher’s exact test comparing distribution of patients with negative or positive COX-2 expression; missing, Nx and Mx values excluded from analysis where applicable.

‡ Chem.: Systemic chemotherapy; RT: Radiation therapy.

### COX2 Expression and Clinicopathological Features

Two-hundred eleven (38%) NMIBCs and 137 (58%) MIBCs expressed COX2 (Tables 1 and 2, respectively), with positive expression defined as a score equal to or greater than the ROC-derived threshold of 0.340 au. Patient and tumor characteristics in the analyzed sample did not differ significantly from the initial SBC/EPICURO study population with the exception of geographic region and tumor size in NMIBC patients (data not shown). The distribution of COX2 positivity was assessed according to established bladder cancer prognosticators including tumor invasion and grade, tumor multiplicity, tumor size and treatment, among others. Demographic factors like age, gender and region were not associated with COX2 expression, nor was the type of primary treatment received by patients (Tables 1 and 2). In NMIBCs, COX2 expression was significantly associated only with T and G; being more prominent in low-grade/risk pTaG1/2 tumors than in high-grade/risk pTa/pT1G3 tumors (p-value<0.0001; Table 1). Further assessment of COX2 distribution in relevant molecular subtypes of UCB [4], revealed a greater proportion of pTaG2 than pTaG1 tumors positively expressing COX2 in low-grade NMIBCs (p<0.0001, subtype 1; Figure S2). COX2 expression did not differ among high-grade/risk NMIBCs (p = 0.075), but a greater proportion of MIBCs positively expressed COX2 than did all high-grade/risk NMIBCs combined (p<0.0001, subtype 2; Figure S2). Only tumor multiplicity was associated with positive COX2 expression in MIBC patients (p-value = 0.008; Table 2).

### COX2 Expression and Prognosis in Bladder Cancer Patients

**Figure 1:**
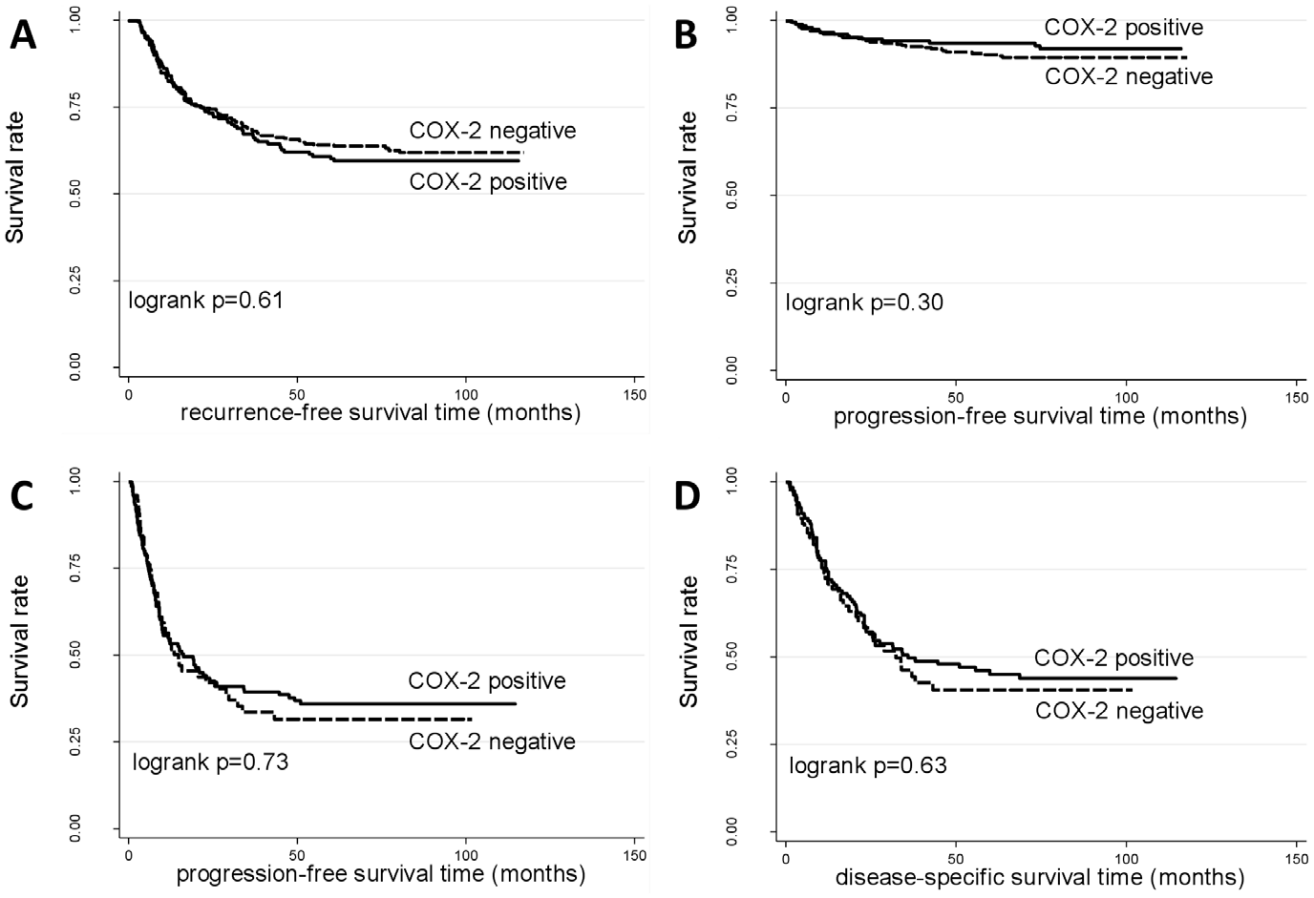
Kaplan-Meier survival curves corresponding to failures in superficial (A, B) and invasive (C, D) tumors for specified prognostic endpoints. Dashed curves: patients with tumors positive for COX2 protein staining; solid curves: patients with tumors negative for COX2 protein staining. Significance values from two-sided logrank test.

We analyzed the association of COX2 expression with tumor recurrence and progression in patients with NMIBCs and with progression and disease-specific survival in patients with MIBCs (Table 3; Figure 1). When considered as a continuous variable in the univariable analysis, COX2 expression was marginally associated with an increased risk of recurrence in NMIBCs (HR = 1.02, 95%CI = 1.00–1.04, p-value = 0.048; Table 3). However, this association disappeared upon multivariable analysis when adjusting for region, gender, tumor stage and grade, multiplicity, tumor size, and treatment. Moreover, COX2 expression was not significantly associated with recurrence in NMIBCs when considered as a categorical variable, neither in the univariable nor multivariable analyses (Figure 1A; Figure S3AC; Table 3). Lastly, no significant association between COX2 expression and progression or survival was observed in patients with NMIBCs or MIBCs, regardless of whether expression was considered as a continuous or categorical variable in non-adjusted or adjusted analyses (Figure 1B–D; Figure S3B, 3D–H; Table 3).

**Table 3:** Analysis of COX2 expression in NMIBCs and MIBCs; univariable and multivariable analyses.

	Univariate COX-regression					Multivariate COX-regression†				
Score Categorization*	Patients, n	Events, n	HR	(95% CI)	P value‡	Patients, n	Failures, n	HR	(95% CI)	P value‡
**Non-muscle invasive tumors**										
**Recurrence** §										
Continuous	556	191	1,02	1,00–1,04	0,048	401	141	1,02	1,00–1,04	0,140
Negative vs. Positive	556	191	1,08	0,81–1,44	0,612	401	141	1,11	0,78–1,59	0,555
Median	556	191	1,08	0,82–1,44	0,583	401	141	1,17	0,82–1,67	0,390
Extreme tertiles	370	127	1,06	0,89–1,27	0,483	268	94	1,08	0,86–1,37	0,510
**Progression**										
Continuous	557	48	0,92	0,84–1,01	0,094	526	43	0,96	0,87–1,05	0,350
Negative vs. Positive	557	48	0,72	0,39–1,33	0,302	526	43	1,38	0,61–3,11	0,434
Median	557	48	0,67	0,38–1,20	0,181	526	43	1,11	0,53–2,33	0,780
Extreme tertiles	371	33	0,71	0,49–1,01	0,059	351	29	0,92	0,54–1,56	0,750
**Muscle invasive tumors**										
**Progression**										
Continuous	216	131	0,99	0,96–1,03	0,617	189	110	0,99	0,96–1,03	0,750
Negative vs. Positive	216	131	0,94	0,66–1,34	0,734	189	110	0,85	0,56–1,29	0,448
Median	216	131	0,97	0,69–1,37	0,869	189	110	0,89	0,60–1,32	0,560
Extreme tertiles	144	85	0,92	0,75–1,14	0,464	128	75	0,90	0,70–1,15	0,410
**Disease specific survival**										
Continuous	216	110	1,00	0,97–1,04	0,908	187	89	1,01	0,97–1,05	0,730
Negative vs. Positive	216	110	0,91	0,61–1,34	0,627	187	89	0,77	0,48–1,23	0,267
Median	216	110	0,94	0,64–1,36	0,726	187	89	0,78	0,50–1,23	0,290
Extreme tertiles	144	68	0,90	0,71–1,15	0,407	126	57	0,78	0,58–1,04	0,090

* Expression cut-points used for categorical variables: "Neg. vs. Pos." - NMIBC/MIBC: 0.340; "Median" - NMIBC: 0.121, MIBC: 0.760; "Extreme tertiles" - NMIBC: (<0.0239, >0.586), MIBC: (<0.270, >2.149).

† Multivariate models adjusted for established bladder cancer prognostic factors as follows: NMIBC Recurrence adjusted by region, gender, tumour stage and grade, # tumours, size of tumours, and treatment; NMIBC Progression adjusted by region, # recurrences, age, tumour stage and grade, # tumours, and treatment; MIBC Progression adjusted by region, tumour stage, treatment, and presence of nodes; MIBC Survival adjusted by region, tumour stage, treatment, presence of nodes, and metastases.

‡ Cox proportional hazards analysis.

§ One patient excluded due to incomplete follow-up record.

### Meta-analysis of COX2 Expression and Bladder Cancer Prognosis

**Figure 2:**
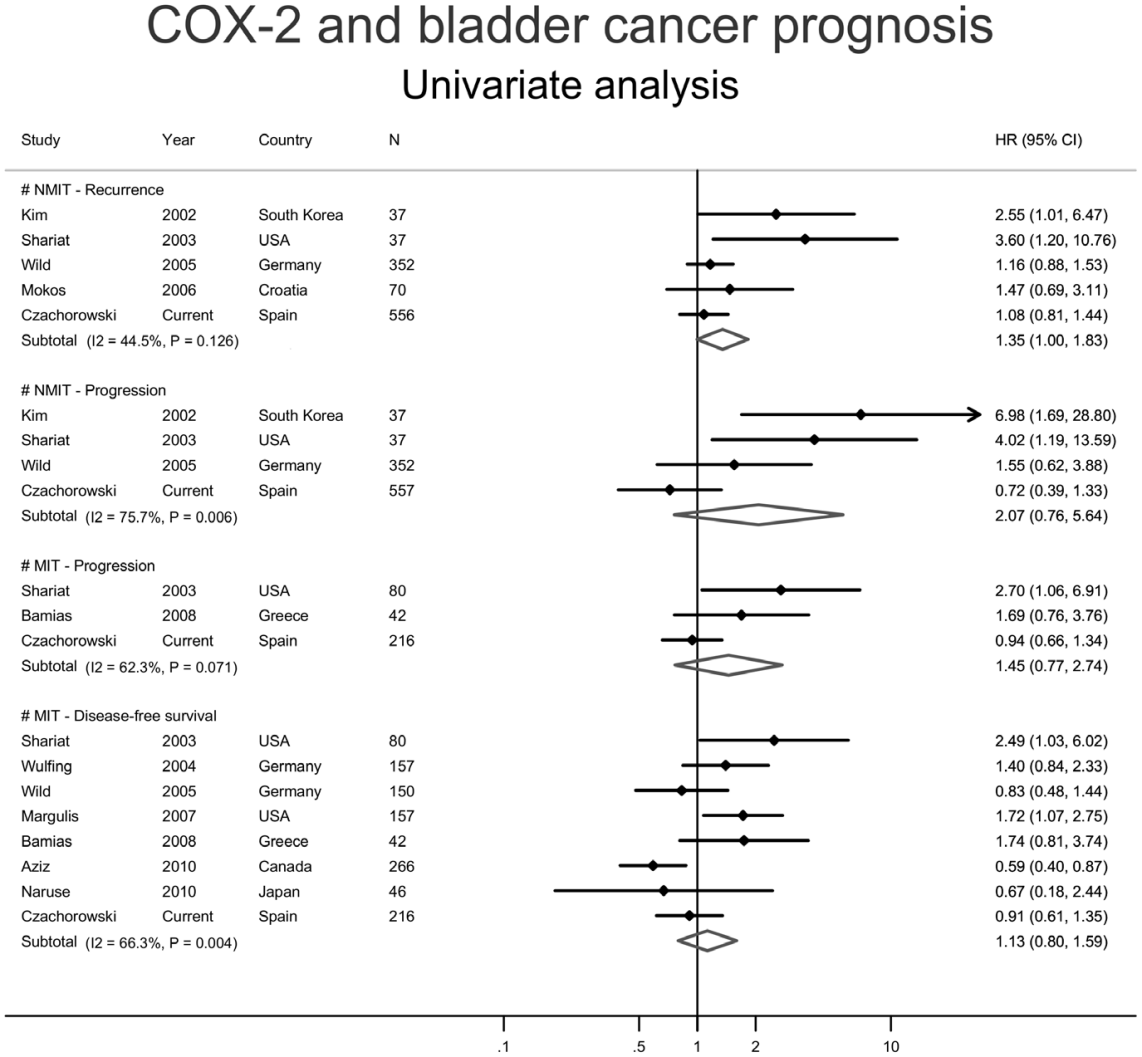
Forest plots from selected univariable studies indicating the risk of reaching the indicated prognostic endpoints in non-muscle invasive (NMIBC; two upper panels), and muscle invasive (MIBC; two lower panels) UCBs in the presence of urothelial COX2 expression.

**Figure 3:**
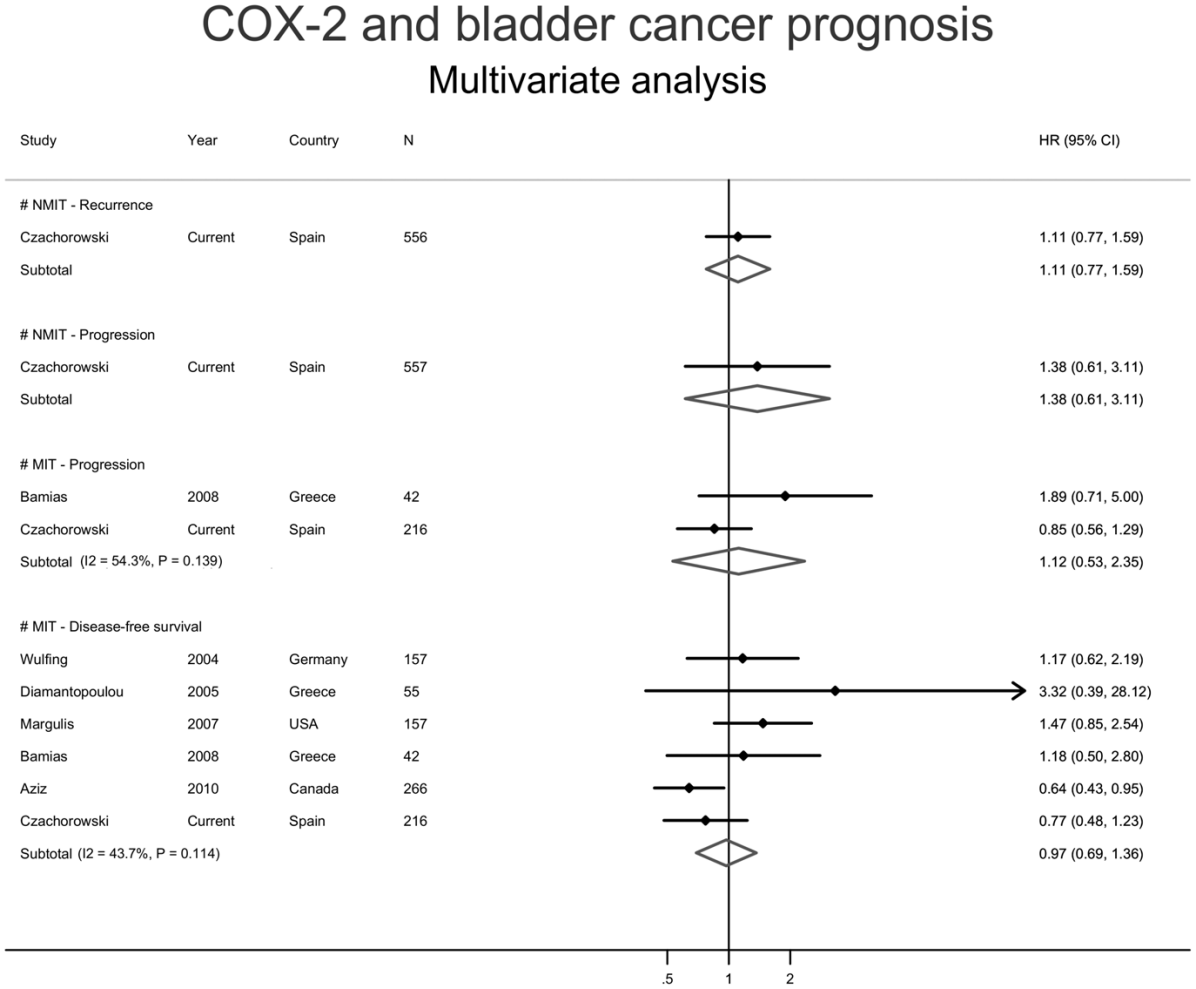
Forest plots from selected multivariable studies indicating the risk of reaching the indicated prognostic endpoints in non-muscle invasive (NMIBC; two upper panels), and muscle invasive (MIBC; two lower panels) UCBs in the presence of urothelial COX2 expression.

Twenty publications on COX2 expression and bladder cancer prognosis were identified through the literature review (Table S1) [12]–[31]. Three of them lacked prognostic data, two overlapped with other larger studies and four included patient cohorts that did not meet the eligibility criteria outlined earlier, leaving 11 evaluable publications [12]–[14], [19], [21]–[25], [28], [29] plus the current study for the meta-analysis (Figure S4). Studies were classified by the tumor subtype(s) they reported on (i.e. NMIBC or MIBC), and whether adjustment for covariates was considered for each prognostic endpoint examined (i.e. univariable or multivariable; Figures 2 and 3). Of the four meta-analyses conducted with univariable data, only the metaHR of the association between COX2 expression and recurrence in NMIBCs showed marginal significance (metaHR = 1.35, 95%CI = 1.00–1.83; Figure 2). This result was not affected by study heterogeneity (I^2^ p-value = 0.13) but exhibited significant publication bias, as evidenced by Egger’s test (p-value = 0.019). The remaining meta-analyses considering univariable data suggested increased, albeit non-significant, risks of tumor progression in patients with NMIBCs (metaHR = 2.07, 95%CI = 0.76–5.64) and MIBCs (metaHR = 1.45, 95%CI = 0.77–2.74), and death in patients with MIBCs (metaHR = 1.13, 95%CI = 0.8–1.59; Figure 2). Notably, the summary effect for progression in NMIBCs and that observed for survival in MIBCs were both significantly affected by study heterogeneity (I^2^ p-values: 0.006 and 0.004, respectively), with the former also significantly influenced by publication bias (Egger’s test p-value = 0.001).

Due to a paucity of published prognostic studies performing multivariable analysis on patients with NMIBCs, we could only address the multivariable meta-association with progression and survival in patients with MIBCs (Figure 3). A small, non-significant increased summary risk of progression (metaHR = 1.12, 95%CI = 0.53–2.35; Figure 3) was observed in COX2 expressing MIBCs that was unaffected by study heterogeneity (I^2^ p-value = 0.139). Similarly, a null summary effect was observed for survival (metaHR = 0.97, 95%CI = 0.69–1.36; Figure 3). This effect was influenced neither by study heterogeneity (I^2^ p-value = 0.114) nor by publication bias (Egger’s test p-value = 0.108.

## Discussion

Despite many published studies, contradictory findings prevail on COX2 expression as an independent prognostic marker in patients with UCB. The current study suggests that COX2 expression is not an independent marker associated with recurrence, progression or survival in patients with UCB.

Using the largest cohort of patients with NMIBCs evaluated for COX2 expression to date, we observed that 38% of these tumors expressed the protein. Other groups have reported frequencies ranging from 53–88%; however, these studies used different COX2 antibodies and expression evaluation techniques and had smaller sample sizes [16], [28], [29], [45], [46]. In accordance with reported results [11], [18], [45] we observed significantly higher COX2 expression in MIBCs (58%) than in NMIBCs. This frequency is similar to that observed in other large, histologically homogeneous studies [21], [28], while groups using heterogeneous cohorts of squamous and transitional cell carcinomas report frequencies different from our own [12], [29]. Collectively, these findings reiterate the importance of homogeneity, or stratification, in tumor marker studies.

The association between COX2 and clinico-pathological characteristics remains a contentious issue in the literature. The majority of studies report an association between COX2 overexpression and advanced tumor invasion and grade, but use heterogeneous populations of NMIBCs *and* MIBCs in their assessments [21], [25], [26], [28], [47]. Given the known disparity in COX2 expression between NMIBCs and MIBCs, an association of this type would be expected in a mixed tumor population. After pooling NMIBCs and MIBCs in our study we also observe a strong significant association between COX2 overexpression and advanced tumor invasion (p>0.0001) and grade (p>0.0001). Notably, several groups report no association between COX2 expression and T and G [14], [29], [45]; especially those working strictly with homogeneous cohorts of MIBCs [12], [23]. Similarly, in our study, COX2 expression did not differ significantly among pT2, pT3 and pT4 tumors (p = 0.896). Interestingly, we observed lower COX2 positivity in pT1 and high-grade/risk NMIBCs, than in pTa and low-grade/risk NMIBCs tumors. This result may seem counterintuitive if grade progression is considered a linear trait and COX2 expression is deemed to increase linearly with T and G. However, there is strong evidence indicating that UCB exists as two molecularly distinct subtypes, with high-grade/risk NMIBCs having a molecular signature more similar to MIBCs than to low-grade/risk NMIBCs [4], [42]. In this respect, we observed that COX2 positivity increased significantly with increasing T and G within each molecular tumor subtype (Figure S2). Shirahama et al. [26] reported a COX2 distribution similar to ours, observing 8% positivity in pT1 tumors and 50% in MIBCs when using whole section staining and a 5% expression threshold. Collectively, these results reiterate the disparity in COX2 expression between NMIBCs and MIBCs first reported by Komhoff et al. [47], and highlight the importance of considering expression within the proper molecular context.

To minimize the effects resulting from selecting an arbitrary expression threshold, we investigated COX2 protein expression as a continuous variable and three categorical variables. Only when considered as a continuous variable in the univariable analysis was COX2 expression found to be associated with a slight increase in the risk of recurrence. The meta-analysis, consisting of five other univariable studies, reiterated this association and showed a 35% increased risk of recurrence in patients with COX2 expressing NMIBCs. However, both effect estimates exhibit only marginal significance, suggesting that the observed associations may be due to chance. Moreover, the association observed in the univariable analysis did not hold after adjustment for conventional prognostic factors of recurrence in the multivariable analysis. Lastly, the summary effect observed in the meta-analysis may have been skewed by two small studies which selected only high risk NMIBCs (T1G3 [19] and Cis [24]). When a sensitivity analysis was performed removing these two studies from the meta-analysis, the association between recurrence and COX2 expression was no longer maintained (metaHR = 1.14, 95%CI = 0.94–1.38). The observed disparity between effect estimates of progression in the present study and the meta-analysis could also be attributed to the inclusion of these two studies. Upon their exclusion, the summary HR showed no association with progression (metaHR = 0.98, 95%CI = 0.47–2.03). These results do not support a role for COX2 expression in NMIBCs as an independent prognostic marker of recurrence or progression.

Several groups have investigated the ability of COX2 expression to predict outcome in patients with MIBCs. Despite wide inter-study variation in methodology, antibodies used, sample size, and adjustment parameters in the case of multivariable analyses, the majority of these studies did not identify any significant association between COX2 expression and progression or survival, consistent with our findings [13], [14], [23], [28], [29]. Shariat and Margulis and their colleagues observed a negative association between high COX2 expression and tumor progression and mortality [21], [25]. However, both studies relied on heterogeneous sample populations which included a small proportion of patients with NMIBCs; potentially accounting for the observed associations given the disparity in COX2 expression between superficial and advanced bladder tumors [45]. In another study, Wulfing et al. reported that high COX2 expression was an independent predictor of poor overall survival in a subgroup of 62 patients with MIBC treated with cisplatin-based chemotherapy [29]. We did not identify any meaningful interaction between COX2 expression and treatment (data not shown), and were unable to replicate their findings in a smaller subset of 39 patients treated with cisplatin (HR = 1.47, 95%CI = 0.48–4.51, p-value = 0.497). Aziz et al. reported a 36% survival advantage associated with increased COX2 levels in a cohort of 266 patients with MIBCs (221 with UCB) that was independent of lymph node status and neo/adjuvant chemotherapy [12]. While we also observed improved survival among patients with COX2 overexpressing MIBCs, this association did not reach significance, consistent with other univariable [23], [28] and multivariable [26] analyses.

Our study had a large sample size, included only incident cases and relied on extensive and accurately acquired follow-up information spanning ten years. Additionally, we used automated scoring of immunostained TMAs, a strategy providing a reproducible assessment of expression that correlated highly with the independent evaluation of a subset of samples by an independent pathologist. COX2 staining was done in one laboratory to avoid heterogeneity in immunohistochemical staining and scoring, and evaluated as a continuous variable in the prognostic analyses to avoid potential bias related to selection of an expression threshold. Moreover, the sample population provides an accurate representation of bladder cancer in the general population as no inclusion criteria were applied in the recruitment process which included a good mix of referral centers and county hospitals. Lastly, the recommendations of the REMARK and PRISMA studies were followed in all of the reported analyses.

Despite these considerations and attempts to accurately quantify COX2 expression only in epithelial cells, the pathologist-trained automated imaging system may have incorporated some immunostained stromal material found on the tissue core, thereby increasing type I error. To reduce potential error we averaged the expression scores from duplicate cores and also explored a method investigated by Henriksen et al. [48] in which the higher score was used (data not shown). Both methods produced similar material associations between COX2 expression and clinico-pathological parameters or HRs. Moreover, adjusted analyses for progression in NMIBCs should be interpreted cautiously given the low number of events in relation to covariates. Also, different patient management practices across recruitment hospitals could increase sample heterogeneity, necessitating the inclusion of both recruitment area and treatment regimen in our multivariable analyses.

The results presented herein focus on COX2 expression levels measured in tumor epithelial cells – only one aspect of the complex interplay between the tumor and the host immune/inflammatory response [49]. The prognostic potential of COX2 (if any) may only be revealed when considered together with other tumoral markers. When investigating several potential prognostic parameters in UCB, Hilmy et al. concluded that systemic factors of the inflammatory response such as levels of C-reactive protein were superior to tumor-based factors such as grade, COX2 expression or T-lymphocytic infiltration [18]. Moreover, in models of cervical cancer, Ferrandina et al. observed that while COX2 expression was mutually exclusive in the tumor and stromal inflammatory cells, high expression in both cell types could be used as an independent marker of poor survival [50]. Future studies investigating the prognostic value of COX2 expression in UCB should take into consideration the multi-factorial and multi-dimensional context of the inflammatory response during carcinogenesis.

The current study is the largest to investigate COX2 expression as an independent marker of outcome in a prospective cohort of UCB patients. These findings, supported by a meta-analysis that included our own data and that from other relevant studies, do not support COX2 tumor cell expression being an independent prognosticator of UCB.

## Supporting Information

Figure S1 **Immunohistochemical staining of COX2 in primary UCBs on TMAs.** Expression was scored as a product of the percentage of epithelial area stained and the staining intensity using automated imaging analysis. A score of <0.340 au was considered negative for COX2 expression, while a score of ≥0.340 was considered positive. Representative sections of a pTaG1 UCB lacking COX2 expression (**A and D**) and a pT2G3 UCB expressing COX2 (**B and E**) are shown. Normal colon tissue was used as a positive control (**C and F**). Upper panels show sections under 100x magnification (**A-C**); lower panels show sections under 200x magnification (**D–F**).
(PDF)

Figure S2 **Distribution of positive COX2 expression in urothelial carcionomas of the bladder classified by their molecular and pathological stage-grade subtypes.** Positive COX2 expression assessed as described in Figure S1. Statistical significance assessed using Fisher’s exact test with a 0.05 significance level. pT1G2 tumors excluded due to low sample size in the current study (n = 11), and a reported tendency to overlap both molecular subtypes.
(PDF)

Figure S3 **Kaplan-Meier survival curves corresponding to failures in superficial (A, B, C, D) and invasive (E, F, G, H) tumors for specified prognostic endpoints and quantiles of COX2 expression.** Dashed curves: patients with tumors expressing COX2 at lower specified quantiles; solid curves: patients with tumors expressing COX2 at upper specified quantiles. Significance values from two-sided logrank test.
(PDF)

Figure S4 **Flow diagram of study selection and inclusion in meta-analysis.**
(PDF)

Table S1 **Main characteristics of eligible studies used in meta-analysis.**
(PDF)
