# Supplementary figures and images for: Cyclooxygenase-2 Expression in Bladder Cancer and Patient Prognosis: Results from a Large Clinical Cohort and Meta-Analysis
Source: PLoS One. 2012 Sep 13;7(9):e45025. doi: 10.1371/journal.pone.0045025 (PMC3441520; doi:10.1371/journal.pone.0045025)

Supporting Figure 1. Immunohistochemical staining of COX2 in primary UCBs on TMAs

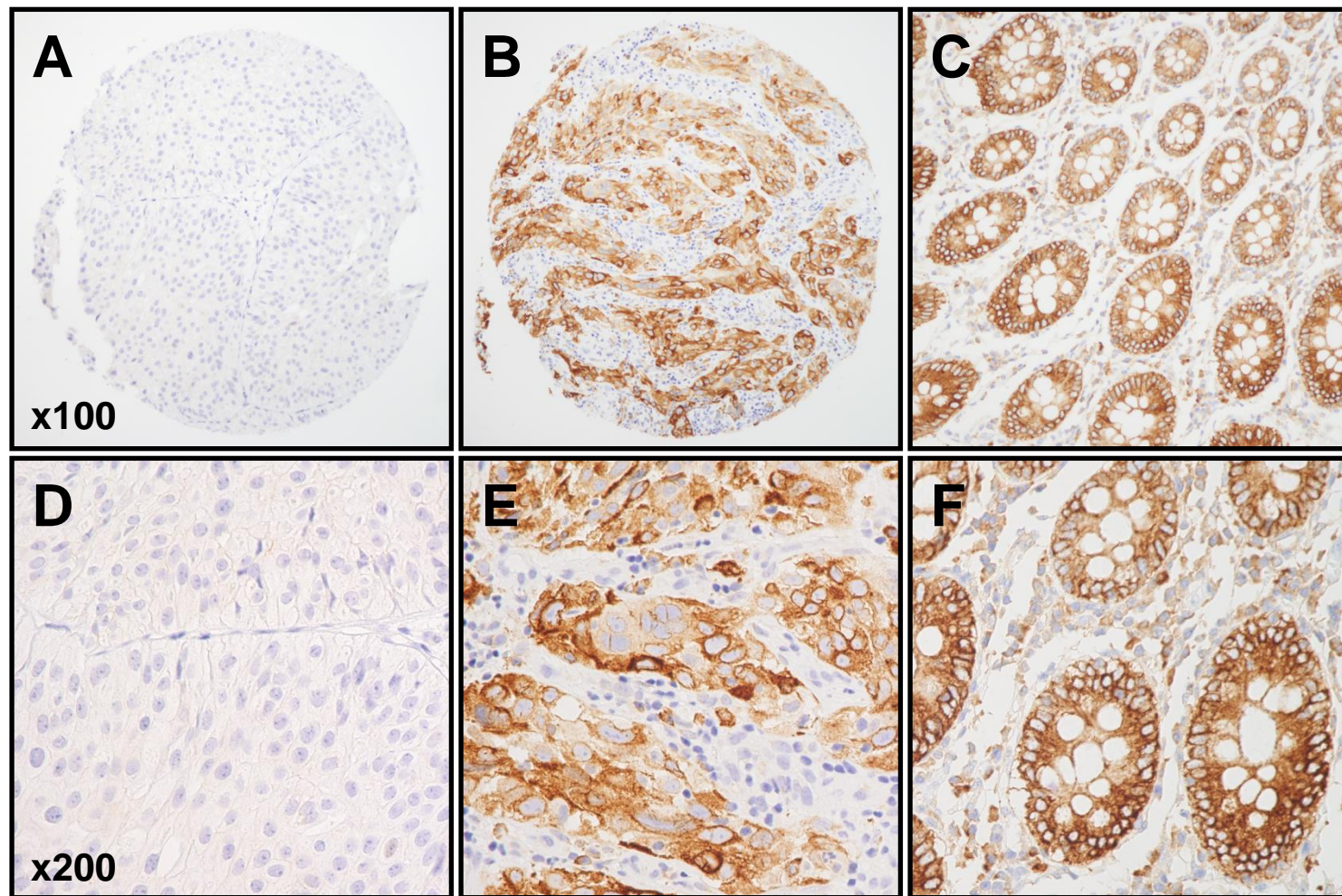

Supplement: Figure S1 — Immunohistochemical staining of COX2 in primary UCBs on TMAs. Expression was scored as a product of the percentage of epithelial area stained and the staining intensity using automated imaging analysis. A score of <0.340 au was considered negative for COX2 expression, while a score of ≥0.340 was considered positive. Representative sections of a pTaG1 UCB lacking COX2 expression (A and D) and a pT2G3 UCB expressing COX2 (B and E) are shown. Normal colon tissue was used as a positive control (C and F). Upper panels show sections under 100x magnification (A-C); lower panels show sections under 200x magnification (D–F). (PDF) [file pone.0045025.s001.pdf]

Supporting Figure 2. Distribution of positive COX2 expression in UCBs

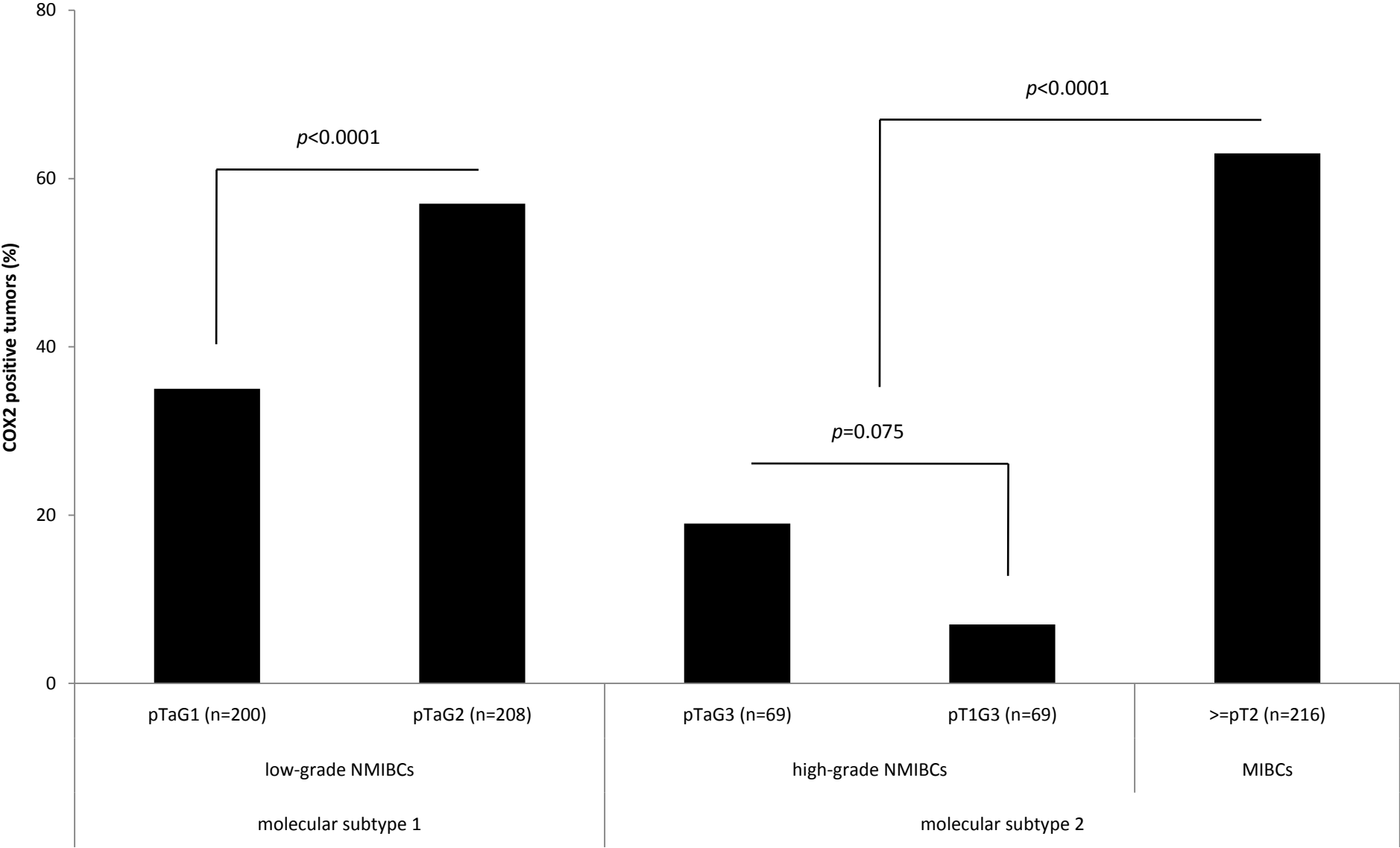

Supplement: Figure S2 — Distribution of positive COX2 expression in urothelial carcionomas of the bladder classified by their molecular and pathological stage-grade subtypes. Positive COX2 expression assessed as described in Figure S1. Statistical significance assessed using Fisher’s exact test with a 0.05 significance level. pT1G2 tumors excluded due to low sample size in the current study (n = 11), and a reported tendency to overlap both molecular subtypes. (PDF) [file pone.0045025.s002.pdf]

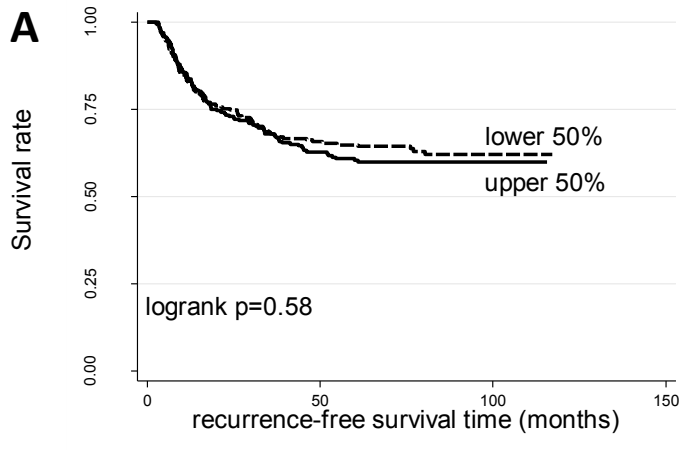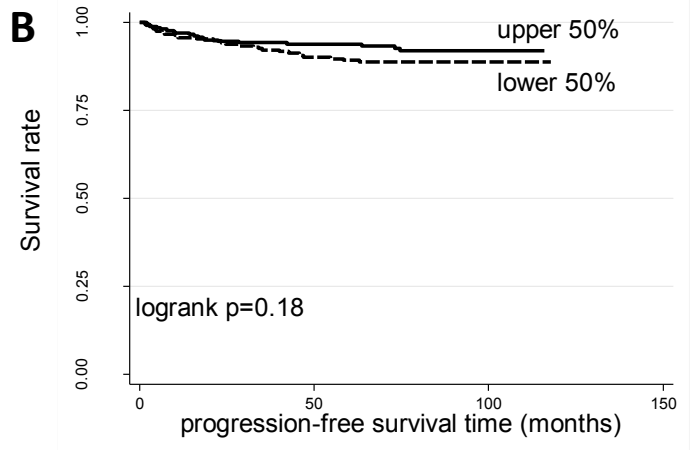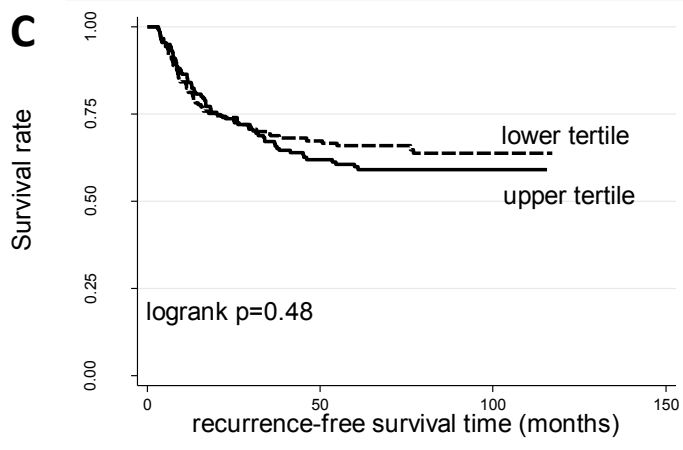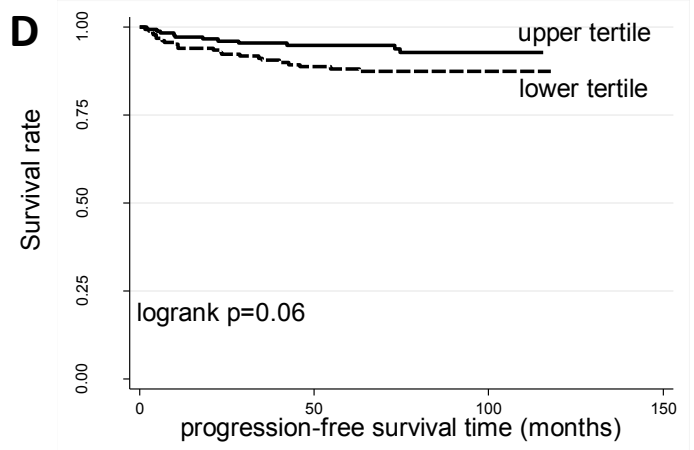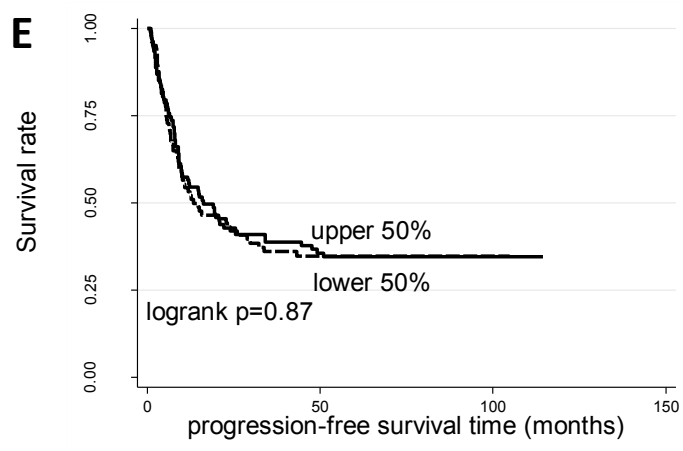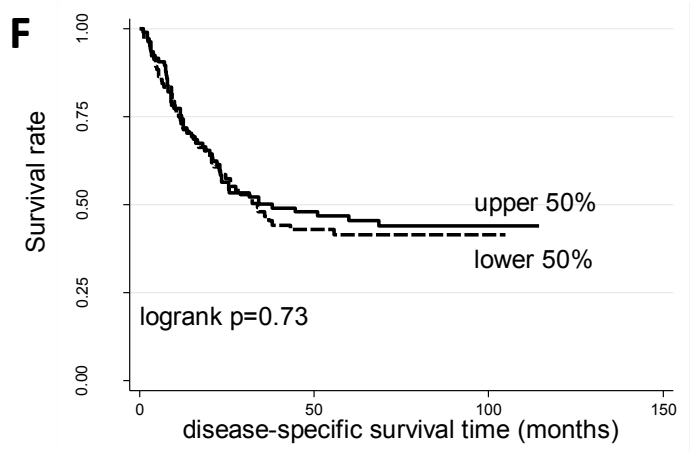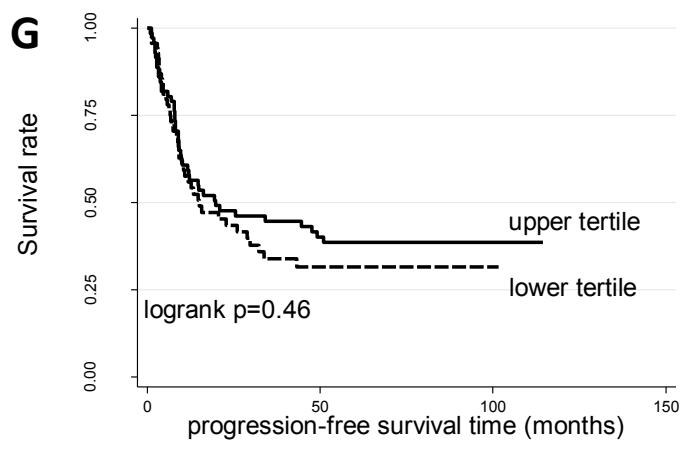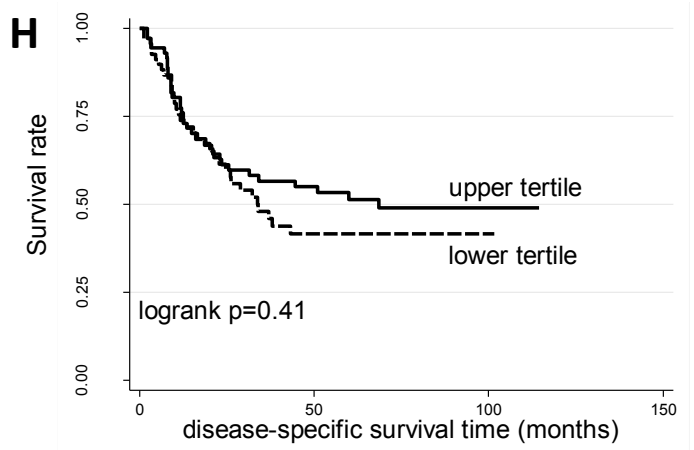

Supplement: Figure S3 — Kaplan-Meier survival curves corresponding to failures in superficial (A, B, C, D) and invasive (E, F, G, H) tumors for specified prognostic endpoints and quantiles of COX2 expression. Dashed curves: patients with tumors expressing COX2 at lower specified quantiles; solid curves: patients with tumors expressing COX2 at upper specified quantiles. Significance values from two-sided logrank test. (PDF) [file pone.0045025.s003.pdf]
