# Supplementary material for: Cyclooxygenase-2 Expression in Bladder Cancer and Patient Prognosis: Results from a Large Clinical Cohort and Meta-Analysis
Source: PLoS One. 2012 Sep 13;7(9):e45025. doi: 10.1371/journal.pone.0045025 (PMC3441520; doi:10.1371/journal.pone.0045025)
Supplement: Figure S4 — Flow diagram of study selection and inclusion in meta-analysis. (PDF) [file pone.0045025.s004.pdf]

Supporting Figure 4. Flow diagram of studies included in the meta-analysis

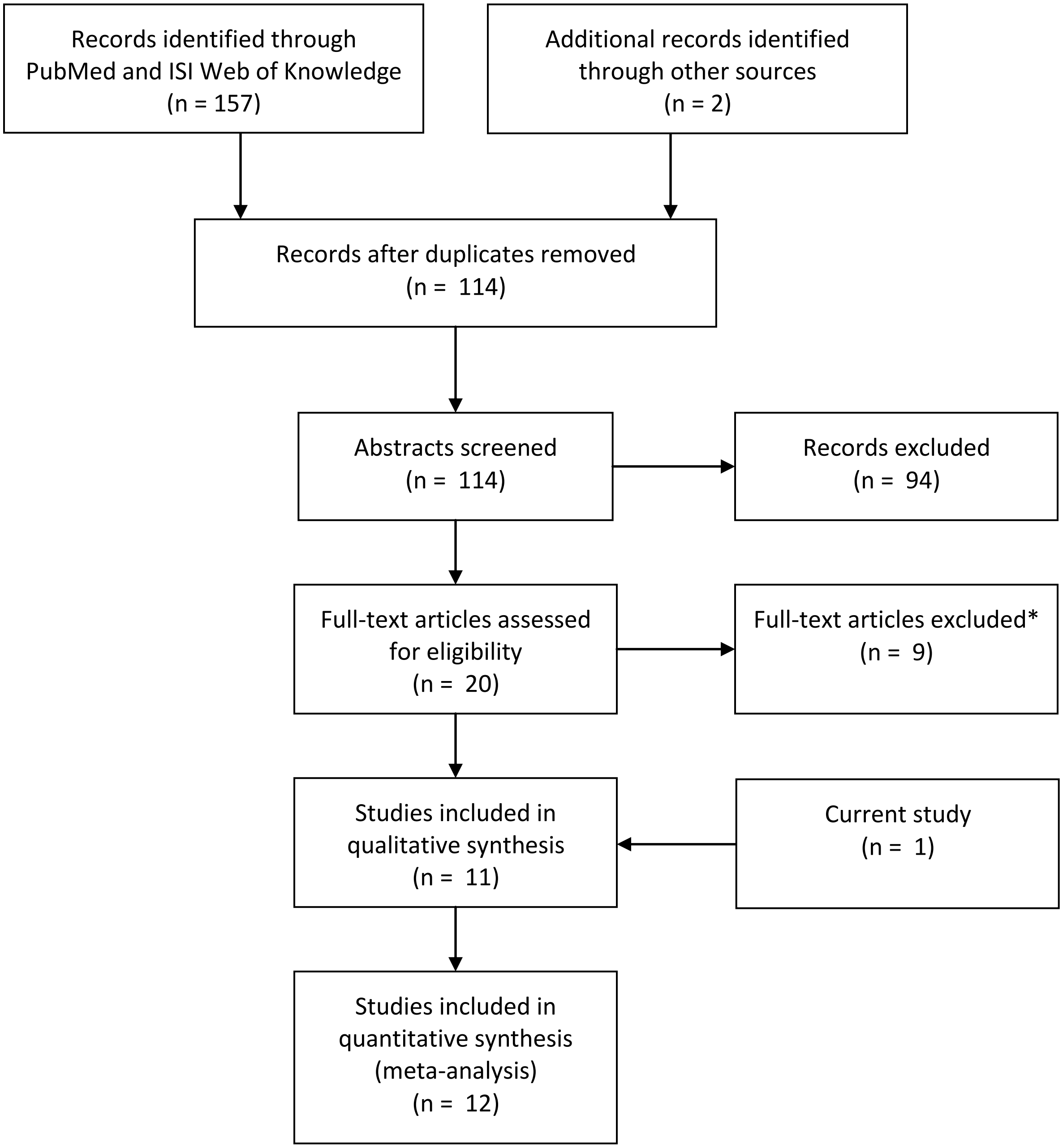

*reasons for exclusion of full-text articles included: no reported prognostic data (n = 3), patient overlap with a larger study (n = 2), pooled analysis on superficial and invasive bladder tumors (n = 2), inclusion of tumors other than UCB in analysis (n = 1), inclusion of non-primary tumors in analysis (n = 1)

Template adapted from: Moher D, Liberati A, Tetzlaff J, Altman DG, The PRISMA Group (2009). *P*referred *R*eporting *I*tems for *S*ystematic Reviews and *M*eta-*A*nalyses: The PRISMA Statement. PLoS Med 6(6): e1000097. doi:10.1371/journal.pmed1000097
