# Supplementary material for: Cyclooxygenase-2 Expression in Bladder Cancer and Patient Prognosis: Results from a Large Clinical Cohort and Meta-Analysis
Source: PLoS One. 2012 Sep 13;7(9):e45025. doi: 10.1371/journal.pone.0045025 (PMC3441520; doi:10.1371/journal.pone.0045025)
Supplement: Table S1 — Main characteristics of eligible studies used in meta-analysis. (PDF) [file pone.0045025.s005.pdf]

Supporting Table 1. Main characteristics of eligible studies used in meta-analysis

First Authour	Year	Country	Patients w/ follow-up	Median follow-up (months)	Histology	Stage	Tissue	Antibody for IHC, dilution	Definition of COX2 positivity	HR Estimation	Evaluable	Notes/reason excluded	Ref.
Aziz	2010	Canada	266	NS	TCC (>75%), SCC	T2-T4	whole section	Cayman, 200	> 5%	HRs/95% CIs	Yes		12
Bamias	2008	Greece	42	72	TCC	pT3, pT4	whole section	SantaCruz, 50	> 10%	HRs/95% CIs; from author	Yes	only bladder tumours analyzed	13
Diamantopoulou	2005	Greece	55	48	TCC	pTa, pT1; pT2-T4	whole section	SantaCruz, 130	>= 10%	HRs/95% CIs; from author	Yes		14
Eltze	2005	Germany	132	88	TCC	pT1; pT2-pT4	whole section	Cayman, 200	> 0, categorical	Surv. curves	No	overlap with Wulfing et al 2004	15
Friedrich	2003	Germany	91	26	TCC	pTa, pT1	NS	Oxford, 200	> 10%	No data	No	insufficient prognostic data	16
Gudjonsson	2011	Sweden	52	37,2	TCC	Ta	TMA	Dako, 5	1, categorical	HRs/95% CIs	No	38% primary tumours in cohort	17
Hilmy	2006	UK	103	60	TCC	Tis, pTa, pT1; pT2-pT4	whole section	Cayman, NS	tertiles examined	HRs/95% CIs	No	pooled cohort of N/MITs	18
Kim	2002	South Korea	37	27	TCC	T1G3	whole section	SantaCruz, 200	> 5%	Surv. curves	Yes		19
Liedberg	2008	Sweden	128	16	TCC	Tis, pT0, pT1; pT2-pT4	TMA	Dako, 5	> 0, categorical	No data	No	no prognostic data	20
Margulis	2007	USA	157	66	TCC	Ta, Tis; T1-T4 (>75%)	TMA	SantaCruz, NS	> 10%	Surv. curves; HRs/95% CIs	Yes		21
Mokos	2006	Croatia	70	24	TCC	Tis, Ta, T1	whole section	Oxford, 50	> 0%, categorical	Surv. curves	Yes	negative vs. positive staining	22
Naruse	2010	Japan	46	NS	TCC	pT2-pT4	whole section	SantaCruz, 20	> 10%	Surv. curves	Yes		23
Shariat	2003a	USA	37	120	TCC	Tis, pT1	whole section	NS, 100	> 10% rec.; > 20% prog.	Surv. curves	Yes		24
Shariat	2003b	USA	80	101	TCC	Tis, pTa, pT1; pT2-pT4 (>75%)	whole section	SantaCruz, 100	>10% of cells	Surv. curves	Yes		25
Shirahama	2001	Japan	108	75	TCC	pT1; pT2-T4	whole section	NS, 200	>= 5%	HRs/95% CIs	No	pooled cohort of N/MITs	26
Tiguert	2002	Canada	172	NS	TCC	MIT	NS	NS, 100	>= 5% of cells	Surv. curves	No	overlap with Aziz et al 2010	27
Wild	2005	Germany	617	39	TCC	pTa, pT1; pT2-4	TMA	Cayman, 5ug/ml	> 0, categorical	HRs/95% CIs; from author	Yes		28
Wulfing	2004	Germany	157	88	TCC (>75%), SCC	Tis, pT1; pT2-pT4 (>75%)	whole section	Cayman, 200	> 0, categorical	Surv. curves; HRs/95% CIs	Yes	negative vs. positive staining	29
Yoshimura	2001	Japan	118	NS	TCC, SCC	pTis, pT0, pT1; pT2-pT4	whole section	"in house", 100	> 0, categorical	No data	No	no prognostic data	30
Youssef	2010	Egypt	315	63,2	TCC (<75%), SCC, AD	pT1; pT2-pT4	TMA	ThermoFisher, 200	> 20%	Surv. curves; HRs/95% CIs	No	TCC (<75%)	31

Abbreviations: AD, adenocarcinoma; HRs/95% CIs, hazard ratios/95% confidence intervals; MIT, muscle invasive tumour; NMIT, non-muscle invasive tumour; NS, not specified; Ref., publication reference; Surv. Curves, survival curves; SCC, squamous cell carcinoma; TCC, transitional cell carcinoma; TMA, tissue microarray.
